# Making sense of Integrated Pest Management (IPM) in the light of evolution

**DOI:** 10.1111/eva.13067

**Published:** 2020-08-20

**Authors:** Kristina Karlsson Green, Johan A. Stenberg, Åsa Lankinen

**Affiliations:** ^1^ Department of Plant Protection Biology Swedish University of Agricultural Sciences Alnarp Sweden

**Keywords:** biological control, crop wild relatives, economic injury level, evolutionary application, evolutionary integrated pest management, pesticide resistance, plant resistance, plant tolerance

## Abstract

Integrated Pest Management (IPM) is a holistic approach to combat pests (including herbivores, pathogens, and weeds) using a combination of preventive and curative actions, and only applying synthetic pesticides when there is an urgent need. Just as the recent recognition that an evolutionary perspective is useful in medicine to understand and predict interactions between hosts, diseases, and medical treatments, we argue that it is crucial to integrate an evolutionary framework in IPM to develop efficient and reliable crop protection strategies that do not lead to resistance development in herbivores, pathogens, and weeds. Such a framework would not only delay resistance evolution in pests, but also optimize each element of the management and increase the synergies between them. Here, we outline key areas within IPM that would especially benefit from a thorough evolutionary understanding. In addition, we discuss the difficulties and advantages of enhancing communication among research communities rooted in different biological disciplines and between researchers and society. Furthermore, we present suggestions that could advance implementation of evolutionary principles in IPM and thus contribute to the development of sustainable agriculture that is resilient to current and emerging pests.

## 1 INTRODUCTION

Pathogens, herbivores, and weeds cause ubiquitous problems for crop production, including 11%–59% losses in yields of the major crops in the world (Oerke, 2006). While resistance traits in wild plants are molded and remolded by natural selection, this evolutionary response has become skewed in agricultural systems since breeding for high yield and good quality has (consciously or unconsciously) removed such traits in crops (Zhan, Thrall, Papaïx, Xie, & Burdon, 2015). Thus, conventional breeding programs have resulted in crop varieties with very low genetic variation in resistance‐related traits (both within and among varieties) and modern agriculture has instead heavily relied on synthetic pesticides (including insecticides, fungicides, and herbicides) to control pests. These synthetic toxins may have adverse effects on humans (Damalas & Eleftherohorinos, 2011; Nicolopoulou‐Stamati, Maipas, Kotampasi, Stamatis, & Hens, 2016) and biodiversity (Geiger et al., 2010; Rundlöf et al., 2015). Moreover, their efficiency has decreased, as numerous pest species have evolved resistance to one or several of the available pesticide compounds (Bass, Denholm, Williamson, & Nauen, 2015; Gould, Brown, & Kuzma, 2018; Ma & Michailides, 2005; Powles & Yu, 2010; Sparks & Nauen, 2015). Thus, pesticides are not always reliable even in cases where they are needed and pesticide resistance has recently been termed a “wicked problem” (Gould et al., 2018).

Since crops are usually grown from seeds bought for each cultivation cycle, they cannot naturally evolve resistance traits against the pests they may be exposed to in the fields. Thus, we need other robust and sustainable strategies to counter crop pests, and we believe that an explicitly evolutionary perspective is needed to develop them. A parallel could be drawn from medicine where short‐sighted use of drugs has led to increased problems of antibiotic resistance in bacteria. The important lesson here is that although antibiotics can defeat pathogens, inappropriate use can promote selection for resistant genotypes of targeted (or other) pathogenic species. Today, there is increased awareness of the need to apply evolutionary theory in medical research (Nesse et al., 2010) not only to mitigate the evolution of antibiotic resistance in pathogens, but also e.g. to avoid development of tumor resistance to therapeutic cancer treatments (Gatenby, Silva, Gillies, & Frieden, 2009).

It was recently suggested that an evolutionary framework is also needed in pest management (Hicks et al., 2018; Neve, Busi, Renton, & Vila‐Aiub, 2014; Thrall et al., 2011; Zhan, Thrall, & Burdon, 2014). Such a framework would allow us to test whether an individual control measure is efficient and predict long‐term consequences of the method for relevant agro‐ecosystems. Here, we develop this concept and argue that an evolutionary perspective is particularly desirable and fruitful for the development of *Integrated Pest Management* (IPM; see also Peterson, Higley, & Pedigo, 2018).

**Figure 1:**
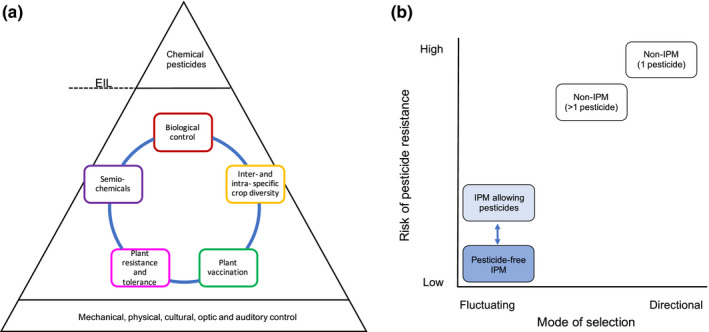
Pest control measures have different selective effects on pests depending on whether they are applied individually or in combination with other measures (i.e., as part of IPM). (a) The IPM pyramid with its largest area of sustainable preventive and curative control methods and a smaller top of chemical pesticide control that could be applied if the Economic Injury Level (EIL) has been reached. In this figure, the base of the pyramid includes, for example, mechanical and physical actions, while the large mid‐section exemplifies ecologically based methods. Modified from Stenberg (2017). (b) A conceptual illustration of the mode of selection that different IPM and non‐IPM approaches may exert on pests and their subsequent consequences for the risk of pesticide resistance evolution. Some of the sustainable pest control measures from the IPM pyramid are likely to drive fluctuating selection on their own, for example, inter‐ or intraspecific field diversity or crop rotation (“temporal intercropping”), while others, for example, biological control or resistance breeding, can change from driving directional selection to diversifying selection through combination with other methods (“Pesticide‐free IPM”). In contrast, pesticide application exerts strong directional selection for resistance in the pests (“Non‐IPM 1 pesticide”). The directional selection could be decreased through combinations or alterations of pesticides ("Non‐IPM >1 pesticide"). However, there may still be a risk for cross‐resistance to develop. EIL could thus be a tipping point for which selective regime that operates in the agricultural fields but the risk to evolve pesticide resistance may be reduced when methods across the pyramid are being used in combination (“IPM allowing pesticides”). Several of the preventive and curative actions could, for example, decrease the potential for resistance development if they are used before pesticides are being applied, for example by increasing gene flow or decreasing the gene pool (Liu et al., 2014; Palumbi, 2001). The different pest management approaches also differ in environmental sustainability, as illustrated with the degree of coloration from white (conventional) to blue (sustainable) in the graph, where IPM without reaching EIL is the most sustainable approach. The arrow represents the range of IPM from completely pesticide‐free to when EIL is reached and pesticides are allowed.

**Figure 2:**
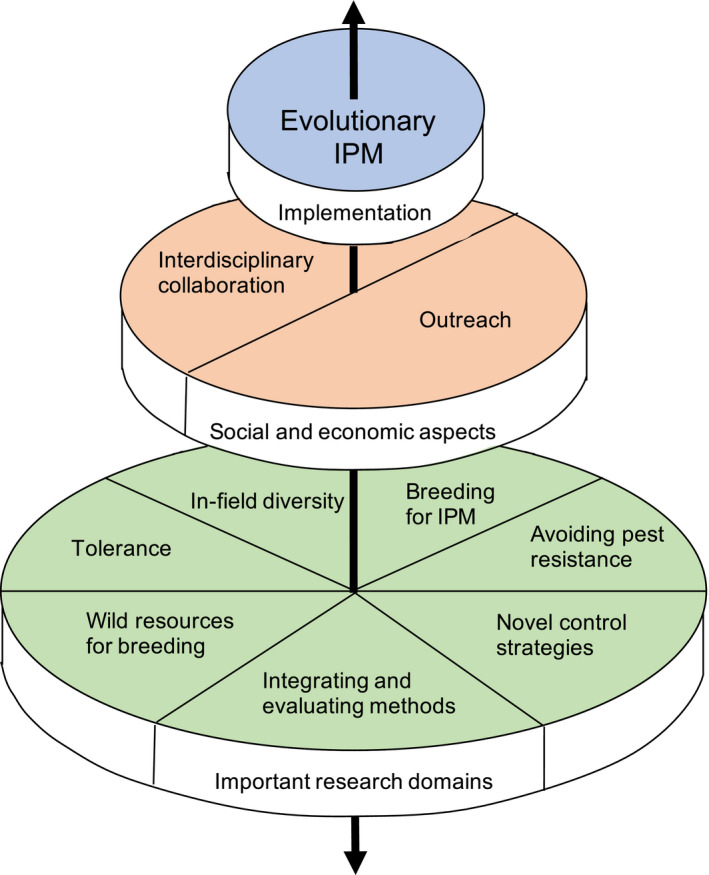
The concept of Evolutionary Integrated Pest Management as presented in the current paper. Implementation of Evolutionary IPM is dependent on research in several domains to develop new approaches for pest management, integrating these methods and evaluating their pest control efficiency as well as evolutionary consequences (green layer). Implementation is also based on social and economic aspects (peach layer), such as a common understanding across disciplines and research funding for interdisciplinary research. Important when developing and implementing the pest management is to convey the significance of an evolutionary perspective to farmers and decision‐makers, as well as incorporating the economic aspects for farmers of the pest management approach. Together, these aspects will facilitate the implementation of Evolutionary IPM (blue layer), which in turn could spur further research as well as an increased understanding in society of the importance of an evolutionary framework (the vertical arrow). Figure inspired by the Sustainable Development Goals “Wedding Cake” made by Azote Images for Stockholm Resilience Centre and presented by Rockström and Sukhdev at Stockholm EAT Food Forum, 2016.

IPM is an approach to combat pests and pathogens using a combination of sustainable methods, thereby becoming less dependent on synthetic pesticides. As opposed to pesticides, the goal of IPM is not to eradicate pests, but to manage them at low numbers below economically injurious levels. Within the EU, it is explicitly prescribed by Directive 2009/128/EC, that all professional plant production within the union must comply with the principles of IPM. IPM could be viewed as a pyramid (Box 1 and Figure 1a) 

where the base layers consist of prioritized preventive methods, and the top layer consists of more curative methods (normally chemical control), which is used as the last resort when other combined actions cannot prevent pests from reaching economic injury levels (EIL; Barzman et al., 2015). An important feature of IPM is the *integration* of different methods and exploitation of their combined, rather than individual, effects (Box 1; Stenberg, 2017). Several strategies may, on their own, retard pests’ evolution of resistance to synthetic pesticides (Palumbi, 2001), for example by decreasing population size or rate of reproduction. In addition, the explicit approach of IPM to combine different control measures may generate fluctuating or balancing selection pressures that further retard evolution of resistance (Liu et al., 2014; Palumbi, 2001; Figure 1). Thus, IPM may in itself be considered a strategy that is more “evolutionarily smart” than applying a single control method that exerts strong directional selection on pests (Figure 1b). However, any single control method may select for resistance in the pests, and there may be preventive and curative methods within IPM that would benefit from knowledge provided by evolutionary research to avoid unwanted evolutionary responses in the pests (Box 1). Management of resistance to pesticides, and other control methods, should preferably also be explicitly included as a component of IPM. The idea of developing an evolutionary framework around IPM is, however, not just to delay resistance evolution in pests, but also to optimize each element as well as the synergies between the different parts in order to increase their efficiency. To confirm that IPM actually is a more evolution smart strategy, the management consequences must be evaluated in an evolutionary framework. As ecological interactions among species usually have shorter timespans than the genetic changes leading to adaptation, although not always (see Catullo, Llewelyn, Phillips, & Moritz, 2019; Jousimo et al., 2014; Koch, Frickel, Valiadi, & Becks, 2014; Turcotte, Araki, Karp, Poveda, & Whitehead, 2017), it is generally easier to observe and study current ecological interactions than their evolutionary outcomes. However, an evolutionary framework is crucial both to understand long‐term consequences and to manage evolutionary‐based problems such as resistance development. Here, we thus suggest how an evolutionary perspective could improve both the management and evaluation of control measures, enabling development of IPM as the sustainable and powerful tool required to counter agricultural pest herbivores, pathogens, and weeds (Figure 2).

Box 1 **Integrated Pest Management (IPM) for dummies**

1 IPM is a holistic approach to combat herbivores, pathogens, and weeds using several methods, while minimizing applications of chemical pesticides. The concept is often illustrated as a pyramid, where various preventive and curative methods form the foundation and chemical control is used only when the economic injury level (EIL) has been reached (Figure 1a).

**Figure 3:**
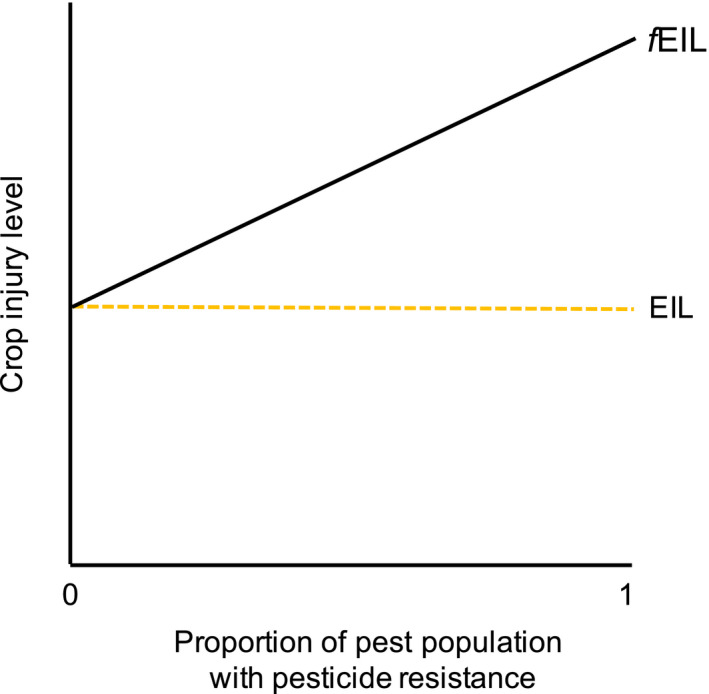
Addressing the risk of resistance development in pests could provide evolutionary‐based support for decisions regarding whether to avoid chemical control or not. Because the risk of resistance development increases with the proportion of resistance alleles in the pest population, chemical control should be avoided for high proportions to lower the risk of resistance development. The economic injury level (EIL), that allows for pesticide application, should thus be flexible and also take into account the potential for resistance development to avoid future pest management problems, *f*EIL (future Economic Injury Level). Yellow dotted line = EIL as a fixed threshold for pesticide application. Black line = threshold for pesticide application depends on proportion of pest resistance (*f*EIL). The curve of *f*EIL does not have to be linear, shown here is a conceptual relationship.

The science of IPM is the systematic study of the compatibility and optimization of simultaneously implemented methods. Such optimization requires an evolutionary perspective which, to date, is lacking.

Commonly used methods that require evolutionary fine‐tuning:

- Chemical control—only to be used as a last option
- Biological control—the use of living organisms to control pests
- Semiochemicals—including insect pheromones and kairomones
- Plant diversity—including intercropping and/or cultivar mixing
- Crop vaccination—including priming and induction of crop defenses
- Plant resistance—including antibiosis and antixenosis
- Plant tolerance—a plant's ability to endure enemy attack without yield loss
- Cultural control—including crop rotation, and watering regime

In addition to these pest controlling measures, IPM programs often include monitoring and forecasting of pest populations, as well as use of decision supporting tools to determine when chemical interventions are necessary. However, evolutionary‐based support tools that provide robust guidance for combining preventive actions have not yet been developed.

As reported here, we have identified several domains where we believe that an evolutionary framework will play an important role in the development of new IPM strategies and predicting their consequences for pest load and yield. We also discuss the challenges and rewards of interdisciplinary research involving both evolutionary biologists and applied researchers, as well as the importance of transferring evolutionary knowledge to stakeholders and decision‐makers.

## 2 IMPORTANT DOMAINS FOR EVOLUTIONARY IPM

### 2.1 Harnessing wild resources for resistance breeding

Intrinsic crop resistance is a fundamental basis for functional IPM. Unfortunately, during the process of domestication, most crops have partly lost important resistance traits (Gaillard, Glauser, Robert, & Turlings, 2018; Whitehead, Turcotte, & Poveda, 2017). A major reason for this loss is that resistance often—but not always (Laine, 2016)—comes at a metabolic cost, causing trade‐offs against other agronomic traits such as yield, size, nutritional quality, and traits that facilitate storage and transport (Evans, 1996). Breeding for high constitutive resistance would thus commonly consume resources from the plant's metabolic budget, leading to reduced possibilities to optimize other traits. Another reason for the loss of resistance is that some underlying traits, such as bitterness and toughness, are undesirable to consumers and therefore actively selected against by breeders. However, an important insight from wild plants is to focus on resistance traits requiring less resources, for example, induced resistance or even less costly priming, that are mainly trigged by pests or “alarm calls” from neighboring plants (Conrath, Beckers, Langenbach, & Jaskiewicz, 2015; van der Ent, Ton, & Corné, 2018) or complementary resistance traits, with lowest‐possible trade‐offs (MacQueen, Sun, & Bergelson, 2016).

Although restoration of resistance (induced or constitutive) is now a major goal of most breeding programs, several problems remain to be solved. First, the available genetic variation in germplasm collections is often too low to allow substantial improvements (Tanksley & McCouch, 1997). A promising solution is to obtain genetic resources from landraces and crop wild relatives (CWRs), because they have not passed through the genetic bottleneck of domestication (Miller & Gross, 2011). Although vast repositories of CWR material have long been available, both ex situ (e.g., gene banks) and in situ (natural populations), enormous resources are often needed to screen and find optimal genotypes. To help breeders focus on the most promising CWR populations, several evolutionary approaches have now been developed. The most widespread method to date is FIGS (“Focused Identification of Germplasm Strategy”), which utilizes the fact that germplasm is likely to reflect the selection pressure of the biotic and abiotic environment in which it evolved (Thormann et al., 2014). Thus, when the geographic distribution of plant resistance to pests likely shaped by natural selection is known, then germplasm can be sampled and screened in a more targeted manner. The FIGS method has been used with various success for major food crops, including wheat (Bari et al., 2012), rice (Vasudevan, Vera Cruz, Gruissem, & Bhullar, 2014), and faba bean (Khazaei, Street, Bari, Mackay, & Stoddard, 2013). However, FIGS is only utilizing one evolutionary factor (i.e., directional natural selection), which limits its precision in cases when other evolutionary factors are important. To improve the resolution, Egan, Muola, and Stenberg (2018) recently developed a new method, which in addition to natural selection also includes proxies of genetic drift and gene flow (e.g., landscape isolation and geographic distance between natural populations). The ongoing progress in this area, making wild genetic resources increasingly available to breeding programs, suggests that the current lack of available traits can ultimately be solved.

The process of restoring or transferring “wild” genetic resources to the breeding material is commonly termed “genetic rewilding” or “inverse breeding,” and different technologies for achieving this goal come with their own set of challenges. Genetic modification (GM) or gene editing (e.g., through Crispr techniques) can in some cases be used when desired traits are coded by few or single known genes (Fonfara, Richter, Bratovic, Le Rhun, & Charpentier, 2016). GM and gene editing techniques are, however, currently strictly regulated and even prohibited in many countries in Europe and elsewhere strongly limiting their use in those countries. Political policies and regulations tend, however, to change over time and may thus be relaxed in Europe in the foreseeable future. In cases when resistance traits depend on many genes, then GWAS‐guided genomic selection and hybridization can be more suitable (Poland & Rutkoski, 2016) and partly help avoid undesired “wild” traits that affect yield and quality. Although genomic selection can speed up the “rewilding” process of domesticated crops, it still does not offer an easy escape from potential difficulties such as incompatibilities and introduction of undesired traits (Dempewolf et al., 2017).

Despite these difficulties, we are convinced that genetic “rewilding” is one of the most important tools toward functional IPM and argue that “rewilding” programs should be developed for all crops that have lost valuable genetic resources for resistance.

### 2.2 Adding tolerance traits

Plants’ responses to antagonists may include not only resistance, but also tolerance, that is, the ability to restrict the harm or fitness reduction caused by a given pest load (Råberg, Graham, & Read, 2009). Resistance traits in plants impose selective pressures promoting traits in pests that help them to overcome the plant defenses and thus reduce their effectiveness. In contrast, plant tolerance is not expected to have negative effects on pests’ fitness and may therefore avoid selection for counter‐adaptations in the pest (Rausher, 2001 but see Vale, Fenton, and Brown, 2014). Increasing tolerance in plants might thus be a more sustainable approach, which does not exacerbate problems in the future through antagonistic evolution of pests (Peterson, Varella, & Higley, 2017).

However, very little is known about the genetic or physiological mechanisms of tolerance or how tolerance against a specific pest will be affected by other plant stresses (Koch, Chapman, Louis, Heng‐Moss, & Sarath, 2016; Peterson et al., 2017). In addition, there is a possibility that tolerance may lead to increases in the spread and populations of pests, which has been explored theoretically (Vale et al., 2014), but rarely studied empirically. Studying tolerance also appears to be complicated by inconsistency of definitions (Castro & Simon, 2016). In addition, it may be difficult to quantify tolerance, for which estimates of reaction norms between pest load and yield or fitness for a number of plants of the same genotype are recommended rather than estimates obtained from observations of a single plant (Råberg et al., 2009). Moreover, it may be important to evaluate effects of combining tolerance and resistance, as it is argued that natural selection should favor either high levels of either defense strategy or intermediate levels of both (Fornoni, Nunez‐Farfan, Valverde, & Rausher, 2004). Thus, to maximize the potential for using tolerance in pest management, there are several opportunities for evolutionary biologists and ecologists to contribute their expertise.

### 2.3 In‐field diversity: increasing genetic diversity in space and time

Genetic diversity is not only needed to optimize traits of individual crop plants, but can also be utilized spatially to optimize intra‐ and interspecific plant diversity within fields. Unfortunately, intensive monocultures of crop species and genotypes (cultivars) are common features of modern agriculture and they exert strong directional selection on pests to overcome control measures. Increasing plant diversity in agricultural landscapes, for instance by cultivar mixing or intercropping, will shift directional selection for particular classes or genotypes of pests with the highest fitness to diversifying selection for pest polymorphisms (Karasov et al., 2018; Zhan et al., 2014), thereby extending the efficiency and durability of existing control methods. Such evolutionary effects on pests may, however, depend on whether specialization incur costs on them, and the efficiency could thus differ between insect pests and pathogens where pathogens often adapt to their hosts through gene‐for‐gene evolution (Brown & Tellier, 2011). Intercropping may furthermore decrease population size of pests that are specialized on either of the cultivated crops, which could slow down pest evolution.

There are several interesting reports on the ecological and productive benefits of increasing in‐field diversity. For example, a recent study showed that growing up to six potato cultivars together in the same field reduced infection by the late blight pathogen *Phytophthora infestans* and increased yield (Yang et al., 2019). Another study found that volatile interactions among barley cultivars reduced aphids’ host plant acceptance, and some combinations of cultivars were better at suppressing the aphids than others (Dahlin, Rubene, Glinwood, & Ninkovic, 2018). In push–pull systems, different plant species are intercropped to simultaneously lure pest insects away from the main crop, suppress weed populations, and improve soil fertility. This has been useful in the control of stemborers (Khan, Midega, Hooper, & Pickett, 2016; Midega, Bruce, Pickett, & Khan, 2015), suggesting that it is another strategy to enhance in‐field diversity that has interesting ecological benefits for crop protection. A challenge with increasing the spatial genetic diversity may, however, be how to harvest if different plant genotypes differ in growth rate and time when they reach maturity. Such factors should be important to address when breeding for IPM (Section 2.4) but could also be tackled by development of machinery and working procedures at the farms.

Temporal genetic diversity can also be promoted, by altering the plant species or genotypes cultivated in a field through crop or cultivar rotation. Such rotations could have important effects on soil‐borne pathogens through plant–soil feedback (Mariotte et al., 2018) and on herbivorous insects that may not be able to move to new fields (Hederström, 2019). Thus, increasing in‐field diversity in both space and time may be important for pest management and could also be used to decrease pesticide resistance evolution in pests (Box 2). Although there are numerous studies on the evolutionary effects of fluctuating selection and environmental heterogeneity in the wild (Kerwin et al., 2015; Robinson, Pilkington, Clutton‐Brock, Pemberton, & Kruuk, 2008), and although agricultural practices in general impose large evolutionary impact (Turcotte et al., 2017), there are still knowledge gaps on the evolutionary effects of diversifying strategies (see Gould, 1991). Evolutionary ecologists could thus make important contributions by evaluating the selective effects on pests from increased in‐field diversity, thereby improving predictions of evolutionary consequences of management regimes incorporating these strategies.

Box 2 **Evolution of Pesticide Resistance**

1 The first indications of pesticide resistance were reported more than 100 years ago, when Melander (1914) asked “Can insects become resistant to sprays?” following observations that efficiency of sulfur‐lime treatment had declined. Since then, numerous cases of pesticide resistance have been detected, commonly within a decade of the introduction of a new substance to the market (Palumbi, 2001), which can lead to substantial costs (Hicks et al., 2018). Despite this pattern, and the very early observation of risks for resistance development, until recently conventional agriculture has focused on developing new toxins to control pests instead of applying them in a manner that reduces the risk of selecting for resistant pests. Evolution of pesticide resistance is affected by genetic variation, strength of selection, and gene flow. The establishment of resistance is a two‐step process: emergence of resistance alleles, followed by a selection phase in which resistant mutants spread in pest populations. The risk for resistance evolution could therefore be decreased, for example, by limiting the gene pool through other curative actions before pesticide application or by allowing gene flow from susceptible individuals in untreated areas. This has been utilized in resistance management strategies, by, for example, usage of refugia, that is, untreated areas where susceptible individuals may persist and then spread and mate with resistant individuals to slow down resistance evolution (e.g., Tabashnik & Carriere, 2015). The strength of directional selection could also be decreased by varying or combining pesticides with different chemical bases or modes of action; however, there are indications that this in fact may select for multiresistant species (Hicks et al., 2018). The evolution of resistance is complex and may depend on pesticide dose, whether resistance alleles are dominant or recessive, whether or not refugia are available and the size of them, and ecological factors (Carrière, Crickmore, & Tabashnik, 2015; Gressel, 2009; Haridas & Tenhumberg, 2018; Mikaberidze, Paveley, Bonhoeffer, & van den Bosch, 2017; Takahashi, Yamanaka, Sudo, & Andow, 2017). However, companies producing pesticides still often recommend high doses at all times (Lindell, 2017). This may conflict with advice researchers and decision‐makers give to growers (Lindell, 2017) and lead to adverse use of pesticides that continues to increase risks for resistance evolution.

It may also be possible to increase fluctuating selection by varying IPM methods in space and time (see e.g., Box 2 on the use of refugia for resistance management). Different IPM strategies could, for example, be varied both among fields and years to further decrease the risk for pests to develop resistance to control methods. Herein is, however, a challenge that there in reality are a limited number of IPM strategies, and thus, there is a need to develop new control methods (see Section 2.6).

### 2.4 New aims for plant breeding

In the application of IPM, we may have to reconsider current plant breeding programs and shift the objective from solely maximizing yield to multiple goals (Weiner, 2017). We have already mentioned the urgent need to improve genetic diversity in plant tolerance and resistance, but crops required for IPM may also need to thrive in environments that differ from conventional agricultural fields. Crops cultivated under IPM may, for example, be intercropped with companion plants that attract beneficial insects (Quinn, Brainard, & Szendrei, 2017) or improve soil condition (Xiao et al., 2019), and they must be able to co‐exist and produce sufficient yields without out‐competing each other. It has therefore been argued that we need to breed our crops explicitly for IPM (Lamichhane et al., 2018), for example, by selecting varieties that are adapted to intercropping or the applied plant protection strategies. Breeding for resistance in general requires evolutionary knowledge on host–parasite interactions (Brown, 2015). To breed for IPM, we must furthermore understand, and exploit, the complex intra‐ and interspecific interactions involving plants within agro‐ecosystems. Plants have, for example, capacities to detect nearby neighbors and respond to their presence, identity, or health status by altering growth and reproductive patterns or initiating defense strategies (Kong et al., 2018; Ninkovic, Rensing, Dahlin, & Markovic, 2019). The directions and magnitudes of such responses may depend on the genetic similarities of the component plants (Ehlers & Bilde, 2019). In addition, the low plant‐genetic diversity in conventional agricultural fields together with current breeding strategies may have induced the development of selection pressures that differ from those that commonly occur in nature. For example, crop traits may have been modified by group selection (Zhu, Weiner, Yu, & Li, 2019) and incorporation of group selection in breeding schemes has been suggested to enhance altruism and cooperation in crops (Murphy, Swanton, Van Acker, & Dudley, 2017; Weiner, 2019). Deeper understanding of plant–plant interactions within and among species, including competition, plant communication, kin‐selection, and even group selection, may thus be important when designing experiments and selecting crop varieties that are best suited for IPM.

Agriculture is also facing new challenges associated with climate change (Schmidhuber & Tubiello, 2007), which will not only alter abiotic conditions for crops but may also lead to the emergence of new and invasive pests. Furthermore, stress due to change in abiotic conditions may affect plants’ responses to current pests (Atkinson & Urwin, 2012). To meet these challenges, agriculture must be flexible and, for example, crops that tolerate varying conditions may be needed. Phenotypic plasticity**—**the responsiveness of a given genotype to environmental contexts (West‐Eberhard, 2003)**—**could be a heritable trait in itself and thus selected for in breeding programs. Although selecting for plasticity may be difficult, exploiting evolutionary research on phenotypic plasticity and breeding plants with higher degrees of plasticity, for example in response to drought or pests, might therefore be an effective strategy to increase agricultural systems’ resilience in a sustainable fashion (Mangin et al., 2017; Marin‐de la Rosa et al., 2019). Furthermore, epigenetic mechanisms that mediate heritable environmentally induced changes in gene expression could also be exploited by breeders to increase crops’ plasticity (Gallusci et al., 2017).

Breeding for IPM and for an unpredictable future may be challenging. For example, to understand the consequences of breeding for plasticity in different traits simultaneously will require knowledge of correlational selection, pleiotropic effects, and costs of plasticity. In addition, it is crucial to understand and, if possible, mitigate the potential negative trade‐offs between, for example, plant resistance and yield, or how the phenotypic variation in crop traits of importance for consumers (e.g., color, size, and taste) is affected by increased plasticity. In some instances, it may be more feasible to utilize genes from CWR’s (Section 2.1) than to breed for new varieties. However, with quantitative genetics we may also draw conclusions from CWR’s on the underlying genetic architecture of important traits and how plant resistance, reproduction, and stress tolerance vary and covary. Such understanding could inform breeders on possible constraints and trade‐offs that may aid breeding programs.

### 2.5 Avoiding pest resistance to chemical control and other IPM methods

Better evolutionary understanding of the development of pest resistance to pesticides or other control measures may have important implications for resistance risk assessment and management, such as the potential for evolutionary‐based decision support also taking long‐term benefits into account (Figure 3). For example, fungicide resistance commonly evolves from de novo mutations in target site‐encoding genes, while standing genetic variation, in combination with de novo mutations, is usually responsible for evolution of insecticide resistance (Hawkins, Bass, Dixon, & Neve, 2019). Knowledge of this difference is important for resistance risk assessment and understanding of variation among pest taxa in both potential for, and mechanisms of, evolution of pesticide resistance. For example, insects (especially generalist species) may have high adaptive capacities to develop pesticide resistance as they already have mechanisms for metabolizing or detoxifying diverse plant chemicals (Hardy, Peterson, Ross, & Rosenheim, 2018), although this pattern also may depend on other factors (Dermauw, Pym, Bass, Van Leeuwen, & Feyereisen, 2018). Thus, application of an evolutionary perspective may improve understanding of taxa that are likely to develop pesticide resistance, types of pesticides that may be most and least persistently potent, and conditions in which pesticides should not be used, even if the EIL is reached. However, resistance may not only evolve in response to synthetic pesticides but to *any* control measure. For example, pest resistance has developed to insecticides expressed by *Bacillus thuringiensis* (Bt) genes in transgenic “Bt crops” (Gassmann, Shrestha, Kropf, St Clair, & Brenizer, 2019; Janmaat & Myers, 2003) and to biological control (Tomasetto, Tylianakis, Reale, Wratten, & Goldson, 2017). Similarly, pests would naturally be under selective pressure to adapt to resistance mechanisms bestowed by incorporating genes from CWRs into crop plants. Hence, it is important to assess the potential evolutionary adaptation of pests to all kinds of control measures, not only pesticides.

Resistance development is an evolutionary process and should be countered with evolutionary knowledge. There are several possibilities for resistance management following evolutionary principles. For example, the use of refugia or pesticide variation (Box 2) takes advantage of gene flow and fluctuating selection to retard resistance development. Other control measures may also be used before pesticide applications to decrease the genetic variation in pests that could respond to selection (Palumbi, 2001). To some extent, these efforts have been successful; however, there are also studies showing that resistance may evolve despite resistance management (Alyokhin, Baker, Mota‐Sanchez, Dively, & Grafius, 2008; Haridas & Tenhumberg, 2018; Hicks et al., 2018; Tabashnik & Carriere, 2015, 2017). This may be due to that cross‐resistance readily evolves to similar classes of pesticides (Liang, Gao, & Zheng, 2003; Sauphanor & Bouvier, 1995; Yu & Powles, 2014) or that resistance often is caused by polygenic metabolic resistance which could detoxify several different pesticides (Haridas & Tenhumberg, 2018). To develop optimal resistance management, more knowledge is thus needed on the mechanisms behind resistance and the genetic underpinnings. For example, initially resistance was considered to be caused by monogenic resistance alleles, while the importance of polygenic resistance is now increasingly highlighted (Busi, Neve, & Powles, 2013; Haridas & Tenhumberg, 2018). In addition, there may be different genetic mechanisms within a species that causes resistance against the same toxins (Van Etten, Lee, Chang, & Baucom, 2020). There is thus a need to investigate both the resistance variation in nature and the evolutionary response of such polygenic resistance to resistance management.

To predict risks for pests developing resistance to specific control measures, knowledge of pests’ resistance‐related genetic variation and early detection of resistant mutants are important. As monitoring of pest populations is an important step toward deciding upon control strategies within IPM, we advocate development of fast, accurate methods for monitoring resistance alleles within pest populations to enable rapid deployment of optimal control strategies in Evolutionary IPM (Figure 2). With the rapid progress in molecular techniques, future farmers could perhaps send pest samples for resistance screening to enable them to take appropriate informed decisions about the most suitable control methods for their particular fields. Such management will, however, be most feasible for monogenic resistance alleles. Thus, it is necessary to assess the genetic basis of resistance, as well as how this varies between populations, before in‐field monitoring of resistance frequencies is enabled.

### 2.6 Developing novel control strategies

For effective IPM, there are strong needs to develop novel, sustainable control methods. For this, knowledge from several evolutionary research areas could be exploited. One established approach to control pest insects sustainably in IPM strategies is mating disruption (Witzgall, Kirsch, & Cork, 2010), that is, reducing pests’ mating frequencies by spreading sex pheromones that affect their localization of mates. Further exploitation of extensive behavioral, genetic, and ecological research on mating and sexual selection may provide various novel paths to explore to reduce pests’ fitness, for example use of sex conversion genes to create all‐male insect populations (KaramiNejadRanjbar et al., 2018). Furthermore, it was recently shown that mating variation in targeted insect populations should be considered during pesticide application, since sexual selection could increase rates of resistance development (Jacomb et al., 2016). Sexual selection in pathogenic fungi and oomycetes might also warrant consideration (Beekman, Nieuwenhuis, Ortiz‐Barrientos, & Evans, 2016). For example, it has been suggested that effectors, small proteins secreted by pathogens that play important roles in infection processes, may have other functions, such as influencing interactions with other microbes and manipulating the host microbiome (Snelders, et al., 2020). As effectors could influence interactions within the same species, they might also be sexually selected.

Another interesting approach stems from advances in our understanding of microbe–plant–insect interactions. Microbial communities associated with plants can promote nutrient uptake, growth, and host resistance to pathogens (Finkel, Castrillo, Paredes, Gonzalez, & Dangl, 2017) and herbivores (Jaber, Araj, & Qasem, 2018). Thus, high diversity of plant microbiota may be an important element of the plant defense system (Hacquard, Spaepen, Garrido‐Oter, & Schulze‐Lefert, 2017). From an evolutionary perspective, it can be expected that microbes would be selected for a high competitive ability and that positive effects on their hosts are side‐effects, while the host would be selected to exert control over the microbiota (Foster, Chluter, Oyte, & Rakoff‐Nahoum, 2017; Snelders et al., 2020). Learning about how plants can maximize their fitness by constructing and maintaining their microbiome could be important for disease control, for example, by treatments with beneficial microbiota or plant breeding on the capacity to control the microbiota (Pascale, Proietti, Pantelides, & Stringlis, 2020). Moreover, better understanding of the connections between plant innate immunity and the plant microbiome may help to improve the efficacy of biological control agents, which is often highly variable in field conditions, for example, they may reduce targeted crop disease by 4%–90% according to Walters, Ratsep, and Havis (2013).

Use of living biological control agents to limit populations of pests and the damage they cause is not a novel approach, but evolutionary research could help to maintain and improve its efficiency and evolutionary sustainability (Gould, 1991). Since such agents are living organisms, they may evolve in response to the breeding regime or laboratory conditions that they are reared in (Kruitwagen, Beukeboom, & Wertheim, 2018). Hence, they could develop traits that may negatively affect introduction of them in the wild or interactions with the pest that they target (Tayeh et al., 2012). With an evolutionary‐based framework, inbreeding or mal‐adaptations of the biocontrol agents could be minimized or selected against before release in the field. In addition, pests may adapt and evolve resistance to biocontrol organisms (Heckel et al., 2007). Breeding of biocontrol agents should thus allow selection for traits that are efficient against the natural variation of defense traits that are present in the targeted pests. Research on the interaction between biocontrol organisms and their preys (see Nygren et al., 2018) will thus be crucial to understand which genes and traits that are important to target in breeding efforts. Breeding of biocontrol organisms may also be integrated with plant breeding, to develop plant varieties that have a synergetic effects on biocontrol (e.g., volatiles that attract the predators) and to breed biocontrol organisms that are well‐adapted to the crop that they should protect (Bottrell, Barbosa, & Gould, 1998; Dotson et al., 2018).

### 2.7 Integrating methods and evaluating combined control effects

To ascertain that a pest management strategy is truly effective and does not lead to resistance development in pest populations, it is necessary to evaluate its possible evolutionary consequences. For example, a common strategy today to avoid or retard evolution of pesticide resistance is to diversify the pesticides used by applying them in rotating schemes or in mixtures (Brent, 2007). However, a recent paper based on long‐term empirical data showed that such pesticide diversification does not necessarily reduce the likelihood of resistance development in pest populations (Hicks et al., 2018), indicating a need to thoroughly investigate evolutionary consequences of management strategies that at first may seem likely to decrease resistance development.

A cornerstone of IPM is the integration of several different control measures. It may be less likely that pests evolve resistance to multiple control methods, targeting various aspects of their biology, than to evolve resistance to multiple pesticides (see Liu et al., 2014). However, there are clear needs to assess the combined effectiveness, evolutionary consequences, and the optimal intensity of each control measure within IPM. For this, evolutionary modeling will be a powerful tool, which can play key roles in formulation of optimal IPM programs and maximization of their efficiency. For example, modeling has already been widely used to explore resistance evolution of pests (Bourguet et al., 2010; Stratonovitch, Elias, Denholm, Slater, & Semenov, 2014) and fine‐tuning resistance management strategies (Haridas & Tenhumberg, 2018). Theoretical modeling may also be useful to predict the spread and establishment of emerging pests and to explore the potential of different crop genotypes in IPM under varying agricultural conditions. Modeling efforts may also incorporate ecological and economic factors, to address pest population growth and economic damage (Menegat, Jack, & Gerhards, 2017). Preferably, pest management should be designed to keep the populations below EIL to avoid severe short‐term economic yield losses, but also take into consideration future possibilities for continued use of effective pesticides when needed (e.g., instead using future EIL, *f*EIL, by including risks of resistance evolution, Figure 3).

To assess the evolutionary consequences of integrated control methods, it is furthermore important to understand how traits, and the underlying genetics, in different species (e.g., crop, pest and biocontrol organism) will respond to selection. However, knowledge about the underlying genetics and evolutionary potential of traits of importance for IPM appears to be particularly scarce, for example plant vaccination, plant tolerance, plant–plant competition, and biological control, and thus requires more attention. Evolutionary outcomes can rarely be predicted from observations and experiments at a single time‐point but experimental evolution has high capacity to assess effects of selective pressures across generations (Kawecki et al., 2012). The vast experience among evolutionary biologists in performing controlled experimental evolution in the laboratory or in studying contemporary evolution in the wild, should be utilized to develop and refine robust, efficient control methods. For example, because pathogens may develop resistance to biological control organisms, the use of experimental evolution could be valuable for detecting biocontrol strains, or combinations of strains, that have more durable effects on plant pathogens.

## 3 INTERDISCIPLINARY COLLABORATION AND OUTREACH

### 3.1 Interdisciplinary collaboration

We believe that cross‐disciplinary efforts involving evolutionary biologists and applied researchers would be synergistic and generate both interesting and useful results. Agricultural research often focuses on mechanisms or interactions among species, but seldom addresses *why* something has evolved or what evolutionary changes current variation may lead to. Thus, it is oriented more toward ecological and short‐term effects than toward evolution and long‐term effects and would benefit from an evolutionary perspective. Similarly, there may be numerous opportunities for evolutionary ecologists to address fundamental research questions within agricultural contexts. Agricultural landscapes provide spatial patchworks of study sites with controlled selection pressures in terms of pest control, which may be historically recorded for decades, and provide excellent frameworks for evolutionary studies (Baucom, 2019). For example, it has been recently suggested that studying rapid and contemporary evolution, such as development of pesticide resistance, may provide new insights in sexual conflict research (Chapman, 2018).

However, communication between research communities and identification of fruitful contexts for synergistic studies may be challenging, partly due to differences in terminologies and paradigms that may start to form even in undergraduate education. Some examples include yield versus fitness and pest insect versus herbivore, where the former terms are used in agriculture and the latter in evolutionary ecology. These terms have sometimes a similar meaning and sometimes not, for example, fitness = yield when measured as seed production but not when measured as number of tubers. In fact, breeding for increased yield may also have led to decreased plant fitness (Weiner, 2019). Interdisciplinary collaboration may thus require both creativity to identify knowledge and theories that can be used in cross‐disciplinary studies, and ability to translate the knowledge into meaningful ideas for all the researchers involved. To facilitate this, interaction among researchers rooted in multiple disciplines is needed, and we encourage cross‐disciplinary meetings and courses on evolutionary agriculture to spur discussion, which in our experience is very rewarding.

### 3.2 Outreach

In addition to the challenges associated with interdisciplinary communication, conveying the message that an evolutionarily rooted strategy is more sustainable in the long term to decision‐makers and farmers could meet several obstacles. A long‐term perspective and understanding of future consequences of current small differences in selection pressure or the amount of genetic variation may seem abstract for someone outside the field, especially if crops are being damaged by pests at this very moment. In addition, there will likely be low motivation for farmers to use sustainable pest control and crop varieties with a better fit to IPM if this has a negative impact on yield. For example, intercropped fields could be difficult to manage and harvest due to the variety of plant species. It may also be problematic if researchers, advisers, and companies offer farmers conflicting advice and propose radically different strategies (Box 2). As mentioned here, IPM may require a new way of breeding crops and conceptualization of agricultural systems. Better understanding of evolutionary dynamics and how our crops and control methods affect, and are affected by, other selective agents in the system may help to establish the more holistic view of pest management that is required in IPM. Furthermore, to ensure that we do not develop control methods that lead to problematic counter‐adaptations in pests, as evolutionary researchers we must clearly convey the importance of taking evolution into account. We thus emphasize the need for evolutionary ecologists to engage in outreach activities and identify value‐laden words and arguments that attract the attention of practitioners and decision‐makers.

It is essential to bear in mind that if the farmer's yield is severely affected, there may be little motivation to adhere to sustainable and evolution smart management. It is thus important to assess whether trade‐offs between yield and a higher degree of plant resistance actually leads to a lower yield for the farmer following pest outbreaks. Research has for example shown that there could be only small differences on yield between pesticide treatment and sustainable management when estimating the losses over years (Wiik & Rosenqvist, 2010). Other consequences following Evolutionary IPM, for example, an increased phenotypic variation in fruits and vegetables which may make crops less attractive at the market, could be mitigated by information to consumers. Awareness by consumers of the advantages of these crops may lead to changed attitudes and acceptance also of vegetables and fruits that differ from the norm.

## 4 CONCLUSIONS

We suggest that Evolutionary IPM is the most promising approach for developing efficient pest control with long‐term sustainability (Box 3). Insights from theoretical modeling, experimental evolution, and selection studies in the wild will improve agricultural capacities to improve pest management and foresee future consequences of today's control methods. Improving predictive abilities will help the agricultural sector to avoid measures that may eventually lead to worse problems or uncontrollable situations. Thus, we recommend and strongly hope that decision‐makers heed lessons from conventional pest management and resistance development in pests and actively advocate evaluation of evolutionary consequences in the development of new control methods.

Box 3 **Challenges and our suggestions for Evolutionary IPM**

Challenges:

- The effects of evolutionary‐based management on yield is not known
- The potential for resistance development to combined control methods lacks information
- Several areas that could be used to develop new control methods lack knowledge, for example, plant tolerance
- The willingness to apply evolutionary‐based management may be low among farmers

Policy suggestions:

- An evolutionary perspective should be systematically integrated into IPM, and the evolutionary consequences of pest management strategies, alone or combined, should be evaluated
- Funding should be devoted to fundamental research on the evolutionary ecology of pest management, which could be subsequently translated into applications
- Evolutionary theory and perspectives should be incorporated into higher level agriculture, horticulture, and forestry education
- Evolutionary researchers should actively engage in outreach and interdisciplinary efforts to disseminate the need for evolutionary competence and approaches in plant protection

Research suggestions:

- Various fields of evolutionary biology and ecology should be surveyed to find aspects to exploit in the development of new crop protections strategies that could be implemented in IPM
- Rewilding programs based on evolutionary approaches should be developed for crops that lack genetic variation for resistance
- The potential for increasing pest tolerance in crops should be explored
- The selection pressures and evolutionary consequences of ecological strategies to increase in‐field diversity in space and time should be investigated
- An evo‐eco perspective should be taken into account to breed plants that are adapted for IPM
- Control measures should be developed and applied in an evolutionarily informed manner to decrease risks for resistance development
- The efficiency of IPM should be evaluated through evolutionary modeling and experimental evolution

## 5 GLOSSARY

Antibiosis: defense that is detrimental for a pest (or other organism).

Antixenosis: defense that deters pests (or other organisms) by affecting their behavior.

Auditory and optic control: pest repellence using noisemakers and visual repellents.

Biologicals: pesticides produced by naturally occurring compounds from living organisms.

Bt crops: genetically modified plants that produce insecticidal proteins encoded by genes obtained from the bacterium *Bacillus thuringiensis*.

Cross‐resistance: resistance to several pesticides due to a common resistance mechanism.

Cultural control: agronomic practices, including crop rotation, timing of sowing and harvesting, and intercropping, and optimization of irrigation and fertilization to reduce pest growth.

Curative actions: actions to eradicate pests and remedy their effects, in contrast to preventive actions, which are taken before pests attack.

Dose dependency: strength of selection that depends on the applied dose of a pesticide.

Economic injury level (EIL): the smallest amount of injury that will cause yield loss equal to pest management costs.

Evolutionary IPM: the integration of evolutionary theory into the development and evaluation of integrated pest control strategies.

*f*EIL: future economic injury level, a threshold based on both injury levels and resistance level in the pest population—see Figure 3.

Genetic rewilding: restoration or transfer of plant resistance (or other traits) to modern breeding material using genetic resources in crop wild relatives, land races, or wild populations of a cultivated plant species.

IPM: Integrated Pest Management—see Box 1.

Modes of action: how a pesticide affects the targeted pest.

Pests: here, herbivores, pathogens, and weeds.

Phenotypic plasticity: differential phenotypic expression of a given genotype depending on the environment.

Physical and mechanical control: modification of, for example, physical barriers and mechanical force or manual labor to remove, exclude, kill, or disarm pests.

Resistance evolution: see Box 2.

Sexual selection: variation in mating success depending on between‐sex mate choice and within‐sex competition for mates.

Wicked problem: a complex problem that is exceptionally difficult (or impossible) to solve due to interacting and changing uncertainties and competing interests.

Crop wild relatives: wild plant species that are closely related to a domesticated crop. They are repositories of genetic variation for breeding improved crop varieties.

Yield loss: the difference between expected and obtained yield.

## CONFLICT OF INTEREST

None declared.

## Data Availability

Data sharing not applicable—no new data generated.
